# Cellulitis in Hajj Pilgrims: Role of Environmental Temperature and Population Size of Pilgrims as a Contributory Factor

**DOI:** 10.7759/cureus.37369

**Published:** 2023-04-10

**Authors:** Ahmed H Badrek-Alamoudi

**Affiliations:** 1 Surgery Department, Faculty of Medicine, Umm Al-Qura University College of Medicine, Makkah, SAU

**Keywords:** dermato-surgery, eco-bio-epidemiology of human diseases, mass religious gathering, temperature, climate changes, international and travel medicine, religious pilgrimage, makkah province, cellulitis, hajj

## Abstract

Background

Cellulitis is a common infection of the skin and subcutaneous tissue. Meteorological and environmental temperatures were previously identified as potential risk factors for causation and the patient’s odds of hospitalization. In this regard, we aim to study the pattern of cellulitis during 10 Hajj seasons and examine the impact of changing seasonal temperatures and overall pilgrim populations as potential risk factors.

Methodology

In-hospital cellulitis was studied within the context of the Hajj. A retrospective review of pilgrim patients coded for cellulitis was undertaken for the Hajj seasons between 2004 and 2012. Possible roles of environmental temperatures, pilgrim population sizes, and ethnicity were examined as potential risk factors.

Results

A total of 381 patients belonging to 42 nationalities were identified, with 285 (75%) males and 96 (25%) females with a mean age of 63 years. On average, cellulitis accounted for 23.5% of general surgical admissions with proportional increases from 2004 to 2012 (r= 0.73, p= 0.016), which significantly correlated with the rise in seasonal temperatures (r = 0.7, p= 0.023).

Conclusions

The findings of this study identified cellulitis as a significant health risk during the Hajj, which is likely to be prevalent in warmer seasons. Our results may assist clinicians in educating Hajj pilgrims of different nationalities about the increased risk of cellulitis during warm seasons and possible predisposing environmental factors of infection.

## Introduction

Cellulitis is a common infection of the skin and the subcutaneous tissue associated with discomfort, erythema, swelling, and warmth of the affected area [1]. Susceptibility to cellulitis is related to microorganism virulence, host immunity status, and environmental factors. A recent systematic review suggested that overall worldwide mortality rates attributed to cellulitis and abscess range from 0.7% to 1.8% [2]. Further studies from Taiwan, Australia, and the United States have suggested that seasonal and meteorological factors play an important role in causing skin infections. Moreover, the odds of hospitalization with cellulitis increase with higher environmental temperatures in a dose-response fashion, with cases peaking in warmer months [3-5].

The Hajj is a Muslim pilgrimage to Makkah and its surrounding holy sites in Saudi Arabia [6]. It is the last pillar of Islam, and it is obligatory to perform at least one Hajj in a lifetime for a Muslim adult. It occurs annually in the month of Dhul Hajj which is the 12th month of the Islamic calendar. Approximately 2.5 to 3 million pilgrims, many in their middle and old ages, from 180 countries perform the rituals for one to two weeks [7]. The Hajj is an epidemiological amplifying chamber [8]. Mass gathering and migration, crowding and congestion, heat, and physical exhaustion are the main risk factors contributing to various disease outbreaks [9,10]. The health of those attending mass gatherings may be at risk from outbreaks of infectious diseases [11]. Given the mismatch in the Hijri-Gregorian calendars, each Hajj season is expected to fall 11 days short on the Gregorian calendar, with environmental temperatures varying depending on whether the Hajj season falls in the winter or summer months. Recorded temperatures during the Hajj range between 37°C and 45°C. This in addition to changes in the number and diversity of pilgrims results in variations in the spectrum of disease outbreaks [12-15]. Dermatologic conditions, whether exacerbations of a preexisting disease or the occurrence of new ones, account for 4.5-5.5% of all diseases seen during the Hajj period [16]. Few reports have suggested that cellulitis incidents account for 1-2% of in-hospital admissions during the Hajj; however, seasonal variations in cellulitis incidents are yet to be established [17,18]. Therefore, this study aimed to retrospectively examine the relationship between the incidence of cellulitis admission across 10 Hajj seasons.

## Materials and methods

Patient selection, demographics, and exclusion criteria

This study was conducted in Al-Noor Hospital, a 550-bed tertiary hospital facility in the city of Makkah with affiliated primary care units in the holy sites at Arafat, Mina, and Muzdalifa, as well as other areas in the greater region of Makkah. Upon review of the research proposal, the Biomedical and Ethical Committee at the Faculty of Medicine, Umm Al-Qura University concluded that ethical approval was unnecessary. Using the International Classification of Diseases, Tenth Revision (ICD-10), adult pilgrim patients coded for cellulitis were retrospectively identified through password-protected electronic records. Search periods were limited to 10 successive Hajj seasons from the beginning of available records (1424-1433 Hijri; 2004-2012). This is a period characterized by an uninterrupted flow of pilgrims due to major infrastructure projects in the Grand Mosque and other holy sites as well as more recently the COVID-19 pandemic. Based on the Hajj-season parameters set by the Ministry of Hajj and Ministry of Health, the season commenced on the first Dhu’l-Hijjah, ending on the 29th Dhu’l-Hijjah (the 12th month of the Islamic calendar). As such, search terms were limited to admission dates during this period. Admissions before and after this period were excluded from this study.

Inclusion Criteria

Analysis was conducted on all adult (aged above 14 years), non-Saudi, pilgrim patients admitted with the diagnosis of cellulitis (based on ICD-10 criteria) under the department of general surgery during 10 successive Hajj seasons (1424-1433 Hijri; 2004-2012).

Exclusion Criteria

Pediatric patients (aged less than 14 years) and those with orbital cellulitis, septic diabetic feet, and postoperative wound infections were excluded. In addition, as the focus of this study was on pilgrims visiting the Kingdom of Saudi Arabia for the purpose of the Hajj, Saudi citizens and local expatriates were also excluded due to the inability to retrospectively distinguish those who were pilgrims from those who were non-pilgrims using current medical electronic records.

Patient’s demographics including, age, gender, and country of origin were noted. Other information was also collated including cellulitis anatomical location, length of hospital stay, types of surgical intervention when required, and treatment outcomes.

Data on the total number of pilgrims were sourced from the General Authority for Statistics, Kingdom of Saudi Arabia (GASTAT KSA) [19]. Ambient Hajj temperatures are based on Saudi Meteorological Office (SMO) records [20] and are defined as the temperature recorded on the day of Arafat (the ninth day of Dhu’l-Hijjah). The data were entered in Microsoft Excel 2020 for further analysis on GraphPad Prism software.

Data analysis

Data were analyzed using the paired t-test (two-tailed) on Prism 5 for Mac OS X software (version 5.0, 1994-2010 GraphPad Software). The chi-square test was utilized to determine the predictive mortality index of age 60 years and above in addition to the male gender. A p-value <0.05 was considered significant.

## Results

The longitudinal seasonal distribution of general surgical admissions in relation to cellulitis admissions, pilgrim population size during the Hajj, and environmental temperatures over 10 Hajj seasons from 2004 to 2012 are shown in Table 1.

**Table 1:** General surgical and cellulitis Hajj admissions, pilgrim population size, and climate temperatures over 10 Hajj seasons from 2004 to 2012. *: The Hijri calendar is 11 days shorter in comparison to the Gregorian calendar. **: Percentage of cellulitis admissions in relation to total general surgery admissions.

Gregorian Year	Hijri year*	Number of pilgrims	Climate temperature (°C)	General surgical admissions	Cellulitis admissions (%)**
January 2004	1424	2,012,074	30.2	197	25 (13)
January 2005	1425	2,164,479	30.2	246	20 (8)
January 2006	1426	2,258,050	30.2	204	18 (9)
December 2006	1427	2,378,636	31.8	153	23 (15)
December 2007	1428	2,454,325	31.8	213	23 (11)
November 2008	1429	2,408,849	35	167	45 (27)
November 2009	1430	2,313,278	35	179	89 (50)
November 2010	1431	2,789,399	35	137	51 (37)
October 2011	1432	2,927,717	39.9	132	50 (38)
October 2012	1433	3,161,573	39.9	142	37 (26)

The geographical distribution of patients admitted with cellulitis is shown in Table 2. A total of 381 patients belonging to 42 nationalities were identified, with 285 (75%) males and 96 (25%) females. The mean age was 63 years with a confidence interval (CI) of 61-64 (minimum = 24, maximum = 92), as shown in Table 3.

**Table 2:** Distribution of patients admitted to the surgery department in Al-Noor Specialty Hospital with cellulitis during the 2012 Hajj season. *: Based on multiple press releases.

Continent/Region	Nationality of pilgrims (pilgrim number for 2012)*	Number (%)
Hajj’s five most populous nations	Egypt (78,000)	31 (8)
	Turkey (313,000)	16 (4)
	Pakistan (180,000)	66 (17)
	India (170,000)	72 (19)
	Indonesia (220,000)	13 (3)
Africa	Central Africa	12 (3)
	East African	9 (2)
	North Africa	24 (6)
	South Africa	6 (1.5)
Americas	North America	3 (0.8)
Asia	Arabian Gulf States	18 (5)
	Central Asia	23 (6)
	China	13 (3)
	The Indian subcontinent (others)	16 (4)
	Iran	4 (1)
	Middle East	28 (7)
	Southeast Asia	14 (4)
Australasia	Australia	2 (0.5)
Europe	Europe	20 (5)

**Table 3:** Male and female cellulitis patients admitted over 10 Hajj seasons from 2004 to 2012 and the mean ages of mortality. CI: confidence interval

Patient’s demographics	30-day mortality rate, number (%)
Male pilgrims	285 (75%)
Female pilgrims	96 (25%)
Mean age	63 years; CI (61-64 years)
Mortality rate, numbers, and (%)	Total: 13 (3.4%); male: 11, female: 2
Mortality mean age	Male: 67 years, female: 66 years

As shown in Table 3, the 30-day mortality rate was 13 (3.4%). Overall, 84% of the deceased were men whose mean age was 67 years.

Disease characteristics and hospital course and outcomes are detailed in Table 4. Anatomical areas affected by cellulitis were recorded in 343 (90%) patients. In total, 73 (19%) patients required surgical interventions, and a further 24 (6%) underwent plastic surgery for skin coverage. The mean duration of hospital stay was four days (minimum = 1, maximum = 24 days), with 304 (80%) patients attaining full recovery, and 63 (17%) absconders and discharged against medical advice. On average, in-hospital mortality occurred six days following admission, with 40% of mortalities occurring at or within the first 24 hours.

**Table 4:** Disease characteristics, hospital course, and outcomes in cellulitis patients. *LOHS, Length of hospital stay

Clinical course	Surgical interventions	Number (%)
Anatomical distribution	Lower limb unilateral	317 (83)
	Lower limb bilateral	14 (4)
	Upper limb	18 (5)
	Abdominal wall	1 (0.2)
Surgical intervention	Incision and drainage	13 (3)
	Debridement	59 (16)
	Amputation	1 (0.2)
	Plastic skin coverage	24 (0.6)
Length of hospital stay	Mean length of hospital stay	4 days (minimum = 1, maximum = 24)
	Single day admission	85 (22)
	Hospital stays beyond four days	185 (40)
Outcome	Full recovery	304 (80)
	Discharge against medical advice	63 (17)
	30-day mortality	10 (3)

Patients from the five most populous (non-indigenous) pilgrim nations, namely, Indonesia, Pakistan, India, Egypt, and Turkey, accounted for 51% of admissions (Table 2). There was no correlation between the number of pilgrims from individual nations and their corresponding cellulitis rates (r = -0.380, p = 0.52).

**Figure 1:**
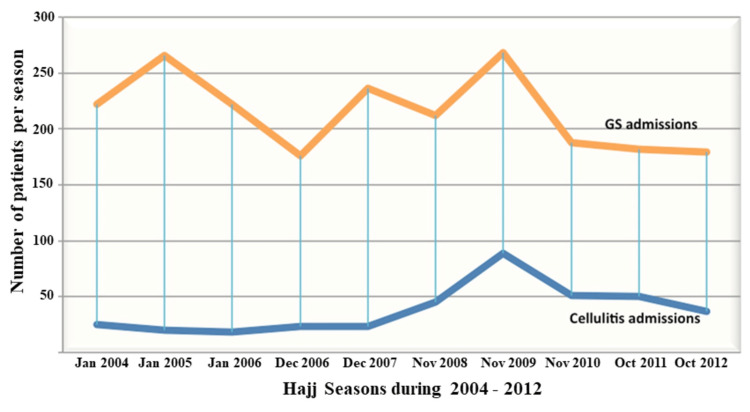
Distribution of general surgical and cellulitis hospital admissions over successive Hajj seasons (January 2004 to October 2012). GS: general surgical

While the proportion of cellulitis cases in relation to overall general surgical admission showed some variability from one Hajj season to another (Figure 1), peaking in the Hajj season of 2009, a regression linear analysis of our data showed a weak but significant uptrend toward an increase as Hajj approached warmer seasons (r^2^ = 0.56, p = 0.026, t-test).

**Figure 2:**
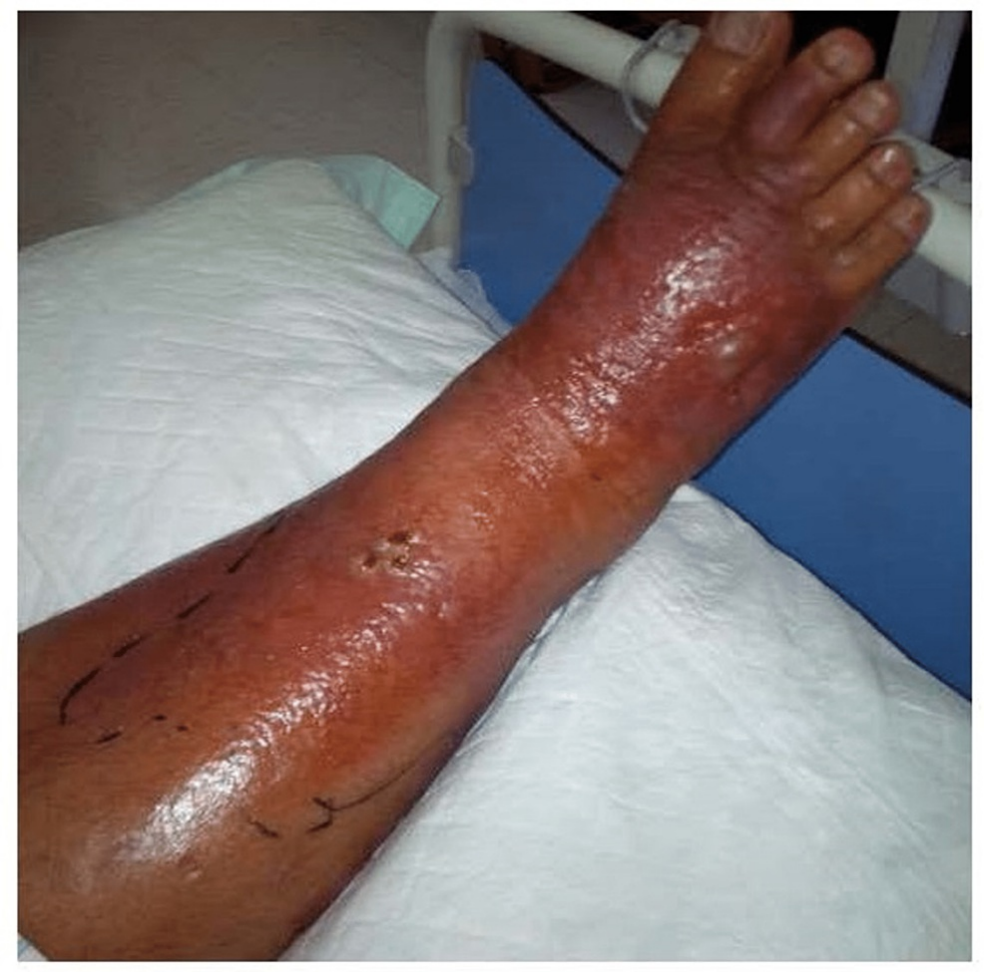
Inflammation due to cellulitis in the lower limb involving the anterior aspect of the leg and dorsum of the foot.

On average cellulitis constituted 23.5% (minimum = 8%, maximum = 50%) of the overall number of general surgical admissions. When compared to general surgical admissions, a significant proportional increase in cellulitis was noted over successive Hajj seasons (r = 0.73, p = 0.016). A representative picture showing inflammation due to cellulitis in the lower limb involving the anterior aspect of the leg and dorsum of the foot is shown in Figure 2.

The official figures by the Saudi Central Department of Statistics and Information (SCDSI) and SMO show an increase in the number of pilgrims from 2 million in January 2004 to 3.15 million in October 2012. Over the same period, the average monthly temperature highs gradually increased from 30.2°C to 39.9°C (Table 1). On examining a possible correlation between the total number of Hajj pilgrims for each season and the frequency of cellulitis in our cohort, our analysis showed a correlation coefficient of 0.46, and this was non-significant with a p-value of 0.25. On the other hand, correlation analysis between the frequency of cellulitis and temperature showed a strong positive and significant correlation (r^2^ = 0.7, p = 0.023).

Table 5 summarizes the type and frequency of cellulitis-associated comorbidities. The most common comorbidity was diabetes mellitus in 22% of patients, followed by hypertension and ischemic cardiac disease in 12% and 4.5%, respectively. Table 6 summarizes the relationship between the length of hospital stay and the number of comorbidities presented in Table 5. Only three or more comorbidities appeared to correlate with longer hospital stays (p = 0.0292, CI = 3.79-9.81). On the other hand, neither the number nor the type of comorbidities had a bearing on mortality rates (Table 6).

**Table 5:** Associated comorbidities in patients with cellulitis.

System	Condition	Number of patients (%)
Psychiatric		1 (0.3)
Neurological conditions	Paraplegia	1 (0.3)
	Cerebral vascular accident	2 (0.5)
	Parkinson’s	1 (0.3)
	Miller Fisher	1 (0.3)
Cardiac	Acute coronary syndrome	17 (4.5)
	Atrial fibrillation	1 (0.3)
	Hypertension	46 (12)
Respiratory	Pneumonia	2 (0.5)
	Pleural effusion	2 (0.5)
	Asthma	3 (0.8)
	Chronic obstructive airway disease	2 (0.5)
	Pulmonary embolus	1 (0.3)
Liver	Cholangitis	1 (0.3)
	Viral hepatitis	4 (1)
	Cirrhosis	3 (0.8)
Renal	Renal impairment and failure	6 (1.6)
Vascular	Raynaud’s	1 (0.3)
	Lymphedema	2 (0.5)
Musculoskeletal	Septic arthritis	1 (0.3)
Metabolic	Diabetes	87 (22)
Total		188 (49

**Table 6:** The mean length of hospital stays in relation to the number of comorbidities.

	Zero comorbidity	One comorbidity	Two comorbidities	Three comorbidities
Number of patients	212	123	36	10
Mean length of hospital stay	4.1	4.2	4.4	6.8
P-value		0.865	0.6735	0.0292

## Discussion

In this retrospective, single-institution, longitudinal review, cellulitis was shown to be a significant health risk during the Hajj, accounting for 8-50% of overall general surgical admissions. In addition, the study yielded interesting observations. In particular, the significant correlation with environmental temperatures, wherein higher admissions were seen in warmer seasons (r = 0.7, p =0.023), is, to our knowledge, an observation not previously made. However, the more recently observed downward trends in admission rates mandate further analysis to confirm current findings, particularly as the Hajj heads toward increasingly warmer seasons. Regarding the correlation of admissions with the size of pilgrim populations, the lack of statistical significance (r = 0.46, p = 0.25) suggests insufficient evidence for crowd size as a risk factor.

Patients’ nationalities underpinning a possible ethnic predisposition are also intriguing. Halpern et al. have shown that white individuals are at a greater risk of developing lower limb cellulitis when compared to their Asian and Afro-Caribbean counterparts [21]. In our case, the non-white ethnic bias as well as the broad ethnic make-up within each nationality precluded such an investigation. However, a recent analysis from northern India found the leg to be the most common site affected [22]. Our results, nonetheless, show a patient’s nationality, in part, mirroring that of the general pilgrim population. Egypt, Pakistan, India, Turkey, and Indonesia, which represent 55% of the total number of non-indigenous pilgrims, were also shown to account for 51% of the total number of admissions. Such patterns are consistent with other medical and surgical Hajj series [23,24]. Yet, when individual nations’ populations were examined against their corresponding admission rates, no clear correlation was demonstrated (r = -0.380, p = 0.52). Whether this is attributed to the limitation in sampling, a true ethnic predisposition, or more complex dynamics involving referral patterns and medical resources within each nation’s mission remains to be investigated.

This study has also provided a comprehensive clinical resource on in-patient cellulitis. Cellulitis is fairly common and is usually treated in outpatient settings [25]; however, our study is in that our analysis comprised only cellulitis patients admitted to general surgical in-hospital care. In our analysis, the mean hospital stay was four days, whereas a study from northern India reported a mean hospital stay of 5.02 days [22]. For the most part, data on demographics, disease characteristics, in-hospital course, and outcomes reveal trends similar to those of non-Hajj cohorts [26,27]. The mean age was 63 years with lower limb cellulitis in the majority of cases (83%), and 49% of patients had a single comorbidity or more, with the most common being diabetes (22%). Successful treatment outcome was shown in 80% of patients, 19% of whom required further surgical interventions over an average hospital stay of four days. Furthermore, 17% of patients were regarded as absconders, pilgrims compelled to migrate to complete the rituals within the constraints of the Hajj period, and in whom treatment outcomes could not be substantiated.

Despite the stark 3:1 male-to-female ratio, this difference was not found to be significant when compared to the expected data (p = 0.06). This is consistent with previous non-Hajj cellulitis series showing marginal variations around an equal distribution [21]. Pilgrim men, unlike women, are obligated to wear chafing garments and compelled to perform certain rituals bare feet. Therefore, it may be conceivable that men are at a greater predisposition to injury and exposure to the elements. The evidence is scarce. However, the Hajj series do show consistent dominance of men [28,29]. Given the lack of risk stratification emanating from inadequate documentation, it is not surprising that the yield of risk factors was unrealistically low (4%). As a prognostic tool, only multiple comorbidities appeared to influence the length of hospital stay with no direct bearing on mortality rates. This contrasts with other series that have shown evidence to the contrary. On the other hand, and in keeping with findings by Perelló-Alzamora et al. [27], the age of 60 years and above was identified as a significant predictor of mortality (p = 0.011). Despite men accounting for 90% of patients within this group, their gender-predictive value was considered to be non-significant (p = 0.26).

Previous research was not focused on seasonal variations in skin diseases by including several Hajj seasons, rather they described analysis of skin and other infectious diseases during one or two Hajj seasons [29,30]. Fatani et al. have described that during the Hajj season of 1998, among the pilgrims treated for skin diseases, cellulitis cases accounted for only 0.4% [18]. Our study is the first to cover 10 Hajj seasons to analyze the incidence of cellulitis.

The single-center, retrospective nature of this study, in addition to the exclusion of indigenous citizens, are obvious limitations that may undermine the validity of current findings. Nonetheless, the longitudinal data have provided an invaluable resource and insight into the possible role of environmental temperatures, crowd numbers, and ethnicity as risk factors in the development of cellulitis and potentially other diseases. These possibilities should be further explored in future prospective multicenter studies.

## Conclusions

This study has identified cellulitis as a significant health risk during the Hajj, which is more likely to be prevalent in warmer seasons and more common in men. Our data on the increased incidences of cellulitis cases with an increase in environmental temperatures when the Hajj pilgrimage season falls in the summer in the Makkah region are in concordance with previously published reports from other countries such as Australia, Taiwan, and the United States. Studies on infectious disease prevention during pilgrimage seasons are scarce, but strict infection control measures are likely to be the cornerstone in mitigating disease spread. However, our data have also provided a comprehensive clinical resource on the course and outcome of inpatient cellulitis establishing multiple comorbidities and advanced age as risk factors for prolonged hospital stay and mortality, respectively. As the number of pilgrims continues to increase every year in Makkah, mass gatherings will continue to increase during the Hajj season. Limiting the spread of infectious diseases at such gatherings requires additional efforts from multiple organizations by learning from past experiences to improve the policies and initiatives for organizing the pilgrimage successfully. Our study may help as a public health message for Hajj pilgrims coming from various parts of the world and to educate pilgrims about the increased risk of cellulitis during the warmer Hajj season.
